# The impact of conventional and organic farming on soil biodiversity conservation: a case study on termites in the long-term farming systems comparison trials in Kenya

**DOI:** 10.1186/s12898-020-00282-x

**Published:** 2020-02-27

**Authors:** John J. Anyango, David Bautze, Komi K. M. Fiaboe, Zipporah O. Lagat, Anne W. Muriuki, Sibylle Stöckli, Judith Riedel, Gladys K. Onyambu, Martha W. Musyoka, Edward N. Karanja, Noah Adamtey

**Affiliations:** 1 International Centre of Insect Physiology and Ecology (icipe), P.O. Box 30772-00100, Nairobi, Kenya; 2 Kenya Agricultural and Livestock Research Organization (KALRO), Nairobi, Kenya; 3 Department of International Cooperation, Research Institute of Organic Agriculture (FiBL), Ackerstrasse 113, P.O. Box 219, 5070 Frick, Switzerland; 4 Zoology Department, Jomo Kenyatta University of Agriculture and Technology, Nairobi, Kenya; 5 Department of Plant Science, Research Institute of Organic Agriculture (FiBL), Ackerstrasse 113, P.O. Box 219, 5070 Frick, Switzerland

**Keywords:** Farming systems, Organic farming, Long-term trial, Soil macrofauna, Termite abundance, Termite diversity, Termite activity

## Abstract

**Background:**

A long-term experiment at two trial sites in Kenya has been on-going since 2007 to assess the effect of organic and conventional farming systems on productivity, profitability and sustainability. During these trials the presence of significant numbers of termites (*Isoptera*) was observed. Termites are major soil macrofauna and within literature they are either depict as ‘pests’ or as important indicator for environmental sustainability. The extent by which termites may be managed to avoid crop damage, but improve sustainability of farming systems is worthwhile to understand. Therefore, a study on termites was added to the long-term experiments in Kenya. The objectives of the study were to quantify the effect of organic (Org) and conventional (Conv) farming systems at two input levels (low and high) on the abundance, incidence, diversity and foraging activities of termites.

**Results:**

The results showed higher termite abundance, incidence, activity and diversity in Org-High compared to Conv-High, Conv-Low and Org-Low. However, the termite presence in each system was also dependent on soil depth, trial site and cropping season. During the experiment, nine different termite genera were identified, that belong to three subfamilies: (i) *Macrotermitinae* (genera: *Allodontotermes*, *Ancistrotermes*, *Macrotermes*, *Microtermes*, *Odontotermes* and *Pseudocanthotermes*), (ii) *Termitinae* (*Amitermes* and *Cubitermes*) and (iii) *Nasutitiermitinae* (*Trinervitermes*).

**Conclusions:**

We hypothesize that the presence of termites within the different farming systems might be influenced by the types of input applied, the soil moisture content and the occurrence of natural enemies. Our findings further demonstrate that the organic high input system attracts termites, which are an important, and often beneficial, component of soil fauna. This further increases the potential of such systems in enhancing sustainable agricultural production in Kenya.

## Background

Stagnant or declining farm productivity in the tropics has been a cause for concern for several decades, mainly due to declining soil fertility and land degradation following the expansion of conventional farming practices [1]. Restoration of soil quality and fertility is a major challenge to local farmers, policy makers and the international agricultural research community. Termites, together with earthworms and ants, are major part of the soil macrofauna and play an important role in enhancing soil quality [2]. However, whereas the effects of earthworms on soil quality have been extensively studied, the effects of termites are not well understood, despite their quantitative importance in many tropical agricultural soils [3].

Within literature there is a distinct dichotomy between that which depicts termites as ‘pests’ and the ecological literature that argues that they play a crucial role as “ecosystem engineers” [4]. Termites (as a pest) often cause partial or total destruction of older crops that have been cultivated for a longer period [5], non-native plants [6] and crops grown during drier seasons [7]. They also damage plants with a high content of lignin and cellulose [8] and crops grown in areas that have been recently cleared or burnt off [9]. The damage can be enhanced by the depletion of alternative food sources for termites or due to loss of their natural enemies [10]. Termites occasionally infest a wide range of host plants in both forestry and agriculture (e.g. maize, cassava, ground nuts, sorghum and sugar cane, rice; and pastures and plantation) where the damage can result in huge losses [4, 11]. Yield losses ranging from 50 to 100% are occasionally reported [12] and it is estimated that globally, termite control costs an estimated $ 20 billion annually [13].

In contrast, results from previous studies have shown that a majority of termite species are encountered in agro ecological systems, but cannot be described as crop pests. Their presence is not correlating with yield losses, instead termites have key ecological functions for soil health [6, 14]. In combination with some agronomic inputs (e.g. mulch), termite presence has been found to improve water permeability [15, 16] and nutrient availability [17]. Similarly a direct link exists between higher termite abundance and enriched organic matter contents [18, 19], increased soil porosity [20], released plant nutrients and stable soil micro-aggregates [21]. Several SSA countries use termite mounds to modify soils for crop production [22], with some communities spreading the terminarium into agricultural lands to achieve maximum crop yields reported [22, 23].

The extent by which termites may be managed to avoid crop damage, but improve soil quality is worthwhile to understand. However, there is some knowledge existing on how biophysical and management factors affect termite abundance, diversity and activity [24, 25], but on the extent how complex farming systems affect termites only little knowledge exists [26, 27]. In Kenya, Long-term Farming Systems Comparisons trials (SysCom; [28, 29]) have been on-going since 2007 at Chuka (Tharaka Nithi County) and at Thika (Murang’a County) to provide evidence on productivity, profitability and sustainability of the different agricultural production systems. In the experiment organic (Org) and conventional (Conv) farming systems are compared at high input levels representing commercial large scale production (high inputs of fertilizer and irrigation) and low input levels representing small holder production, largely for subsistence use (low inputs of fertilizer and rain fed). A detailed study on termites was introduced into the trials to determine how the farming systems (Conv-Low, Org-Low, Conv-High and Org-High) in the long-term experiment influence (i) abundance, incidence and foraging activities of termites (total and casts), and (ii) diversity of termite genus. Furthermore, the study should reveal how the different environmental conditions (trial sites Chuka and Thika), crop patterns (cropping seasons with different crops) and soil depths (substrate, topsoil and subsoil) influence the termite presence, activity and diversity in the various farming systems. From past termite observation in the long-term experiment, our hypotheses was that more termites are present in the farming system Org-High compared to the other farming system. Thus, activity and most probably diversity will be higher in this farming system. Establishing such knowledge can contribute to determining the environmental sustainability of farming systems.

## Results

### The abundance of, and incidence index for total numbers of termites and termite castes

**Fig. 1:**
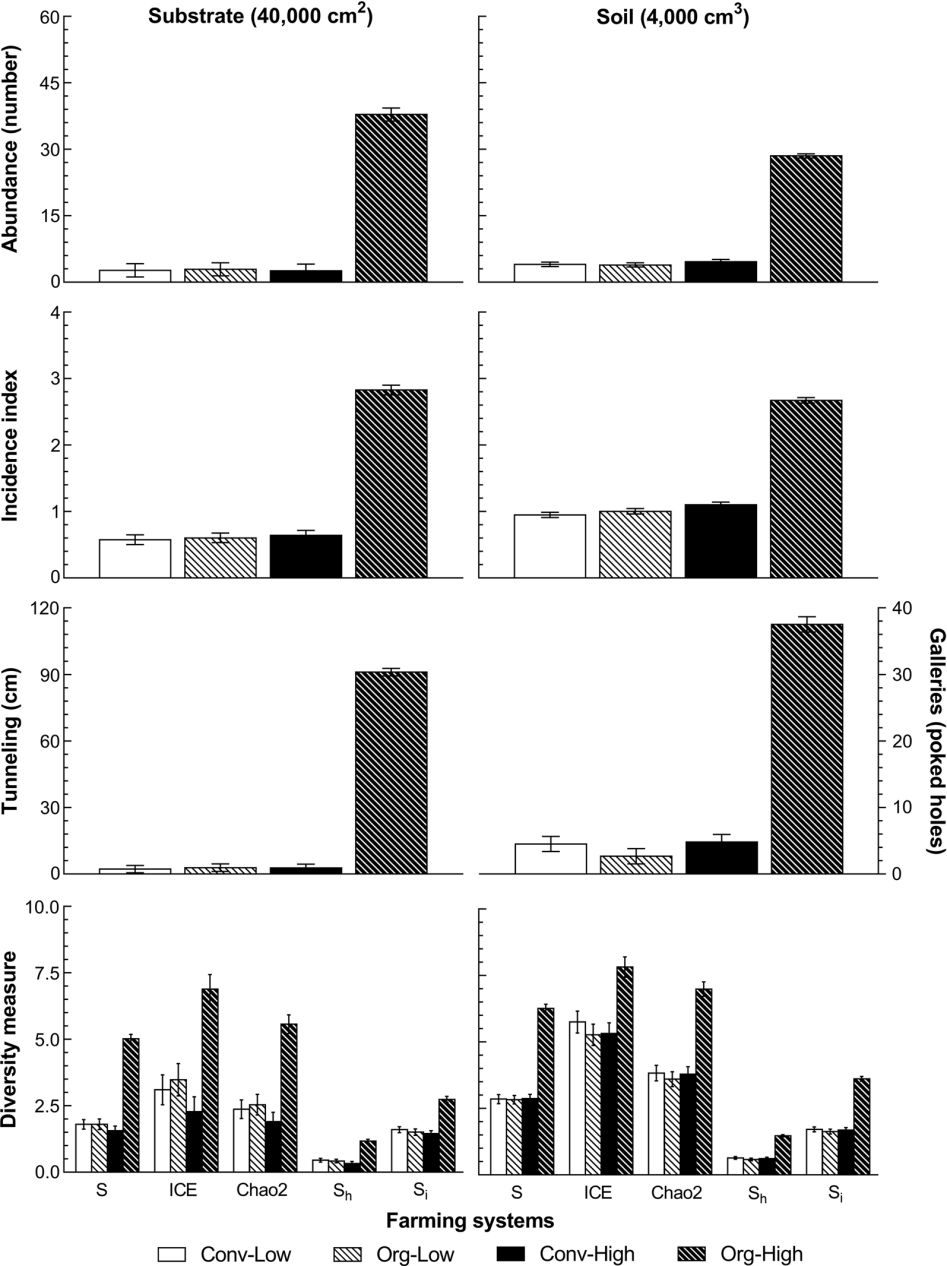
The summarized effect of farming systems on termite number, presence, activity and diversity. The average abundance of, incidence index for, tunneling/galleries activity and diversity measures of the total number of termites in the substrate and soil in long-term farming systems comparisons trials at Chuka and Thika, the Central Highlands of Kenya (error bars: ± standard error of means)

The study sampled a total of over 60,000 termites from the long-term farming systems comparison trials at the two sites. The results revealed general effects of the different farming systems on the average abundance of, and incidence index for termites (Fig. 1). The Org-High farming system had the highest average abundance of total number of termites in the substrate (37.9 ± 1.5 termites per 40,000 cm^2^) and in the soil (28.5 ± 0.5 termites per 4000 cm^3^) over all cropping seasons and trial sites. In all the other farming systems average termite abundance was significantly lower: 13–15 times less in the substrate (value range from 2.6 to 2.9) and 6–7 times lower in the soil (value range from 3.9 to 4.6). The results of the average incidence index showed a similar pattern, with Org-High also having the highest values in the substrate (2.8 ± 0.1 per 40,000 cm^2^) and the soil (2.7 ± 0.1 per 4000 cm^3^) over all the cropping seasons and trial sites. These values were 4.5 and 3 times higher respectively than those found in the other systems (0.6 and 0.9–1.1 respectively).

**Fig. 2:**
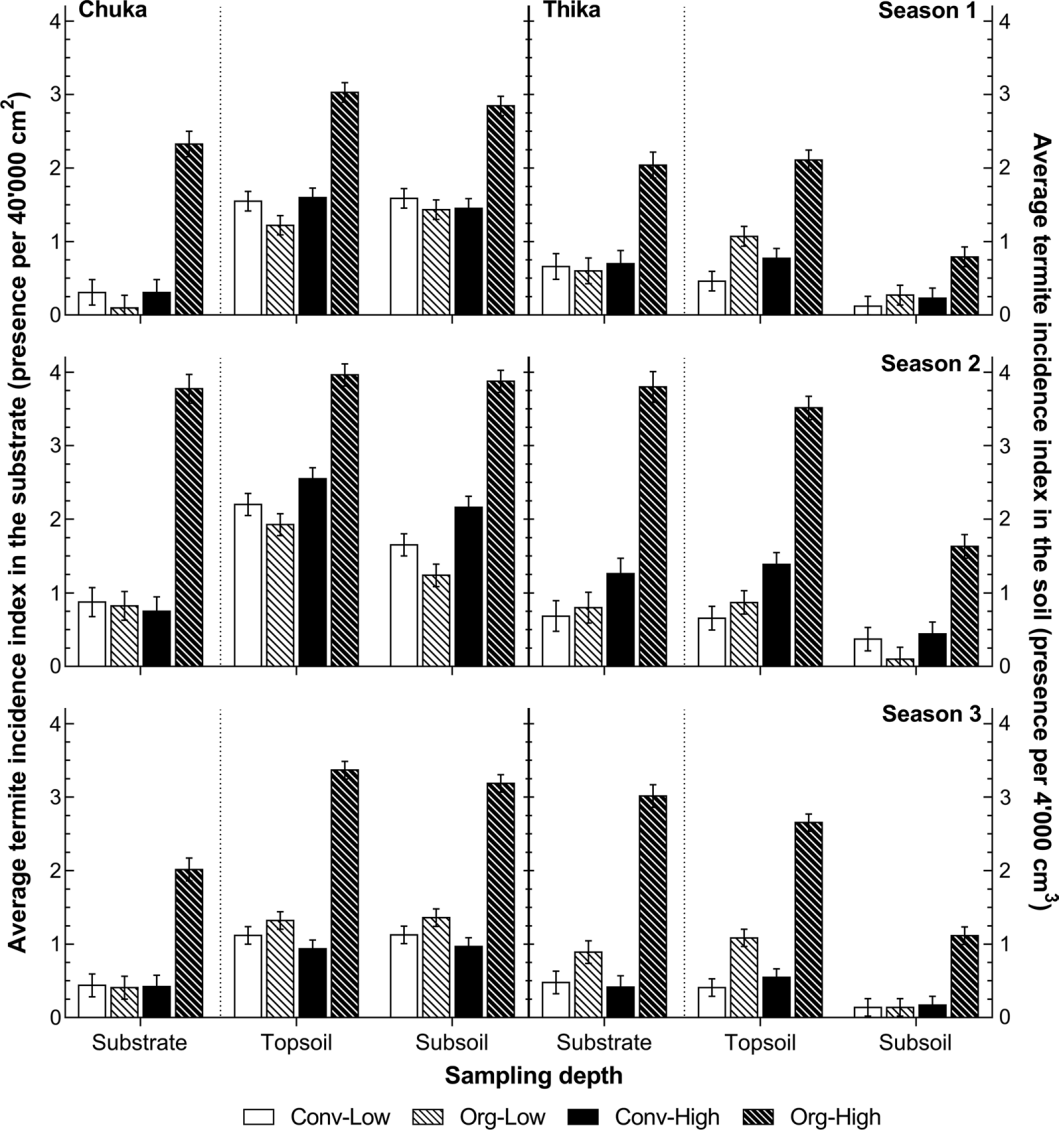
The effect of farming system, trial site, cropping season and soil depth on termite incidence. The average termite incidence index in the substrate and the soil in the 1st, 2nd and 3rd season at Chuka and Thika, the Central Highlands of Kenya (error bars: ± standard error of means)

There were several significant interactions between the factor farming system and the other factors trial site, soil depth and cropping season on the average abundance of and the incidence index for termites (Additional file 1: Table S1). We will further only show the results on average incidence index, because average abundance showed similar results and did not further enhance the knowledge on the influence of farming systems. A graphical representation of the average incidence index for the total number of termites in the farming systems for all cropping seasons, soil depths and trial sites can be seen in Fig. 2.

#### The average incidence index for termites in the substrate

The statistical analysis of all factors revealed a farming system * soil depth * trial site interaction was significant (*p* < 0.01) and showed significantly higher values in Org-High than in the other farming systems in every season at both sites. There were only significant seasonal differences within the Org-High system. At Chuka, Org-High showed the highest values in the 2nd season (3.8 ± 0.2), which was significantly higher than those found in the same system at the same site in the 1st (2.3 ± 0.2) and 3rd season (2.0 ± 0.2). At Thika the highest values in the Org-High system were found in the 2nd (3.8 ± 0.2) and 3rd season (3.0 ± 0.2), which were significantly higher than in the 1st season (2.0 ± 0.2). In addition, the values in the Org-High system in the 3^rd^ season at Thika were significantly higher (3.0 ± 0.2) than at Chuka (2.0 ± 0.2), which showed a difference that was not evident in the other seasons. None of the other farming systems showed such significant differences between seasons or trial sites for the average incidence index of total number of termites in the substrate.

In general, the average abundance of, and the incidence index for, termite castes i.e. workers (2084 individuals found) and immature individuals (9759) in the substrate followed the same patterns as for the total number of termites. This is further confirmed by the significant positive (*p* < 0.001) correlation of the abundance of termite workers (r = 0.99) and immature termites (r = 1.00) with the total number of termites in the substrate (Table 1). The average abundance of termite soldiers in the substrate (997 individuals found) showed a smaller, but nonetheless significant (*p* < 0.001) positive correlation (r = 0.76) with the abundance of the total number of termites in the substrate.

**Table 1:** The correlation of total number of termites and termite casts and activity

(Substrate/soil)	Total	Worker	Soldier	Immature	Tunneling	Gallery^a^
Total	1.00	0.99***/0.99***	0.76***/0.73***	1.00***/1.00***	0.60***/na	na/0.50***
Worker		1.00	0.73***/0.68***	0.99***/0.99***	0.59***/na	na/0.50***
Soldier			1.00	0.73***/0.68***	0.62***/na	na/0.35***
Immature				1.00	0.59***/na	na/0.50***
Tunneling					1.00	na
Gallery						1.00

The correlation (Pearson-r) of termite abundance between the total number of termites, termite castes and tunneling and gallery activity in the substrate (left hand value) and the soil (right hand value) in the long-term farming systems comparisons trial sites at Chuka and Thika, the Central Highlands of Kenya

*na* not applicable

^a^The correlation between tunneling and gallery activity was only calculated for substrate or soil, as the activities were measured at different depths; NB: Significant correlations between total number of termites, termite caste and termite activity are indicated by * (p < 0.05), ** (p < 0.01) or *** (p < 0.001)

#### The average incidence index for termites in the soil

The statistical analysis revealed, that only two of the three three-way interactions were significant for the incidence index of total number of termites (Additional file 1: Table S1). Both interactions showed that termites were significantly more frequently present (i) on the Org-High plots than in all other systems, and (ii) at Chuka compared to Thika. Furthermore, the significant farming system * trial site * soil depth (*p* < 0.001) interaction revealed some further significant differences within the farming systems. At Thika the incidence of termites in three farming systems (Org-Low, Conv-High and Org-High) was significantly higher in the topsoil (1.0 ± 0.1, 0.9 ± 0.1 and 2.8 ± 0.1 respectively) than in the subsoil (0.2 ± 0.1, 0.3 ± 0.1 and 1.2 ± 0.1). Such a distinction did not appear in the Conv-Low system at Thika or in any of the farming systems at Chuka.

The significant farming system * trial site * cropping season (*p* < 0.01) interaction showed no significant differences between the farming systems additionally to the one mentioned above, although the interaction showed significant seasonal and inter-site differences within the two high input systems. The values for Conv-High and Org-High in the 2nd season at Chuka (2.4 ± 0.1 and 3.9 ± 0.1) and Thika (0.9 ± 0.1 and 2.6 ± 0.1) were significantly higher than in the 1st season (1.5 ± 0.1 and 2.9 ± 0.1 at Chuka; 0.5 ± 0.1 and 1.5 ± 0.1 at Thika), and in the 3rd season (1.0 ± 0.1 and 2.9 ± 0.1 at Chuka; 0.4 ± 0.1 and 1.9 ± 0.1 at Thika). No significant differences between the seasons were found within the low input systems.

In the soil (as in the substrate) the average abundance of, and the incidence index for, the termite castes i.e. the termite workers (7800 individuals found) and immature individuals (39,891) followed the same pattern as the total number of termites. This is also confirmed by the significant positive (*p* < 0.001) correlation of the abundance of termite workers (r = 0.99) and immature individuals (r = 1.00) with the total number of termites in the soil (Table 1). The average abundance of termite soldiers (4030 individuals found) showed a smaller, but nonetheless significant (*p* < 0.001) positive correlation (r = 0.73) with total termite abundance in the soil.

### Termite activity: tunneling in the substrate and galleries in the soil

This study also determined termite activity by measuring tunneling (in cm) within substrates, and the numbers of galleries (pocked holes) within soil profiles. Both these determinants were generally affected by the farming systems, as shown in Fig. 1. The Org-High farming system recorded the average highest values for tunneling (87.9 ± 12.4) and gallery activity (36.6 ± 3.3) over all soil depths, cropping seasons and trial sites. These figures were 30–40 and 8–14 times higher respectively than the values for tunneling (range 2.01–2.81) and gallery activity (range 2.58–4.34) recorded within the other farming systems.

As with the other indicators we have described (average abundance and the incidence index), termite activity was significantly affected by other factors, including the trial site, cropping season, soil depths and interactions between these factors and the farming systems (Additional file 1: Table S1). However, no additional trends could be extracted from the data on activity, which would enhance the knowledge on termite behavior. The trends are similar to the already revealed trends in abundance and incidence. This is also shown by the significant positive (*p* < 0.001) correlation between average termite tunneling and gallery activities (r = 0.60 and 0.50 respectively) with total termite abundance in the substrate and soil (Table 1).

### Diversity measures for termite genera in the substrate and soil

A total of 2669 identifiable termite soldiers was found at Chuka and 2358 at Thika, belonging to 9 termite genera, from three sub-families: (i) Macrotermitinae (genera: *Allodontotermes*, *Ancistrotermes*, *Macrotermes*, *Microtermes*, *Odontotermes* and *Pseudocanthotermes*), (ii) Termitinae (*Amitermes* and *Cubitermes*) and (iii) Nasutitiermitinae (*Trinervitermes)*. *Macrotermes* (1641 individuals) and *Microtermes* (1535) were the most abundant and *Ancistrotermes* (36) and *Allodontotermes* (37) the least abundant. *Allodontotermes* and *Ancistrotermes* were exclusively found at Chuka and *Odontotermes* only occurred at Thika.

In general, the highest values for species richness (S), the incidence-based coverage estimator of species richness (ICE), the Chao2 estimator of species richness, the Shannon index (S_h_) and the inverse Simpson index (S_i_) were all found in the substrate, top and subsoil of the Org-High farming system (Fig. 1). The other farming systems generally recorded lower values for these diversity measures. Nonetheless, other factors and interactions between the farming system and other factors, such as soil depth, trial site and cropping season were found to be significant in both the substrate and soil (Additional file 1: Table S2).

In the substrate, all the diversity measures were significantly affected by the farming system (*p* < 0.001) without significant interactions (except for S_i_). The Org-High farming system showed significantly higher values for S (5.02), ICE (6.89), Chao2 (5.57) and S_h_ (1.17) than all the other farming systems. The farming system * trial site interaction was significant for S_i_ (*p* < 0.001) and with Org-High having significantly higher S_i_ values (3.53) than the other farming systems at Thika (which ranged from 1.55 to 1.85). There were significant seasonal differences for S and Chao2 (*p* < 0.001 and < 0.05), which were significantly higher in the 3rd season (3.06 and 3.74 respectively) than in the 1st season (2.25 and 2.65) and also for S in the 2nd season (2.32). The species richness (S), Chao2 and Shannon Index (S_h_) values showed a significant site factor (*p* < 0.001, < 0.05 and < 0.001 respectively) with significantly higher values recorded at Thika (3.02, 3.66 and 0.75) than at Chuka (2.07, 2.53 and 0.43).

Similar patterns were found in the soil. The farming system factor was significant (*p* < 0.001) for all the diversity measures, but significantly interacted with at least one other factor (with the exception of S_i_). For species richness (S) a significant farming system * season interaction emerged, with Org-High having significantly higher values (1st season 5.93, 2nd 6.39 and 3rd 6.46) than all other farming systems in all three seasons of the study. In the Conv-High farming system there were significant differences between the 1st (2.43) and 2nd season (3.64). The statistics for the incidence-based coverage estimator of species richness (ICE) in the soil showed a significant interactions with farming system and all other factors. There was a significant farming system * soil depth interaction (*p* < 0.01) with the values for ICE in the subsoil at being significantly higher in Org-High (8.21) than in Conv-High and Org-Low (4.71 and 4.28). The farming system * cropping season interaction (*p* < 0.05) revealed significant differences in diversity between the farming systems in the 1st and 3rd seasons. In both seasons Org-High showed significantly higher values (1st 7.88; 3rd 8.49) than Conv-High (1st 4.26; 3rd 5.32). In the 3rd season the ICE for Org-High was even higher than the value for Conv-Low (5.14). The last significant interaction, between farming system and trial site (*p* < 0.01), showed no differences between the farming systems at Chuka, but at Thika the ICE for Org-High (7.45) was significantly higher than for all the other farming systems (range 3.44–4.19). The statistical analysis of the Chao2 values revealed two significant interactions: The first, between farming system and soil depth (*p* < 0.05) showed that the Chao2 values in the top and subsoil in Org-High (6.96 and 7.01) were significantly higher than all the other values found in all the other systems (ranging from 2.89 to 4.45). Another interaction, between farming system and season (*p* < 0.05), showed the Chao2 value for Org-High in the 1st (7.10) and 3rd seasons (7.26) to be significantly higher than all the other values found, excluding the value for Org-High in the 2nd season (6.60) (which was not a significant difference in relation to the other seasons’ values). The statistical analysis of the Shannon index S_h_ revealed a significant farming system * soil depth interaction (*p* < 0.01), with all the values for Org-High in the top and subsoil (1.53 and 1.41) being significantly higher than in all other systems at both depths. The inverted Simpson index S_i_ only showed significant effects for farming system and soil depth (*p* < 0.001): Org-High (3.61) scored significantly higher on this criteria than all other farming systems (range 1.64–1.71) and the topsoil values were significantly higher (2.36) than those for the subsoil (1.98).

## Discussion

### The effect of farming systems on termite abundance, incidence index, activity and diversity

The termite populations occurred in varying abundances, incidences and diversities, all of which were consistently affected by the farming systems. Termite populations, activity and diversity were generally higher under the Org-High farming system than under the other farming systems (Conv-Low, Org-Low and Conv-High). The organic based inputs used in Org-High could have been among the main reasons why these plots attracted far more termites. Each season the Org-High plots received FYM-compost (11.3 t ha^−1^), *Tithonia* mulch (5.4 t ha^−1^), *Tithonia* tea (3.9 t ha^−1^) and rock phosphate (364 kg ha^−1^). We hypothesize that the termites were more likely to inhabit these plots as these inputs provided preferred food sources. Refs. [30–32] have all reported similar results, as organic inputs contain cellulose materials that are generally preferred by the termites. They also noted that such inputs release gases that attract termites in large numbers. The cover crop (*Mucuna*) and the mulch material that was used (*Tithonia* in all seasons and rice mulch in the 1st and 3rd seasons) could have further increased the termite population in Org-High plots. A similar effect was also observed in a study by [33]. The lower termite abundance, incidence and activity observed in the other three farming systems was most probably due to their receiving fewer organic inputs and a result of the use of inorganic chemical fertilizers in the conventional systems. Similar results have been reported by [6].

Another possible explanation for the higher termite population, activity and diversity in the Org-High plots could be the irrigation that these plots occasionally received during dry spells. While the Conv-High plots also received irrigation water the Org-High plots were less liable to evapotranspiration from the topsoil and substrate due to the presence of cover crops and mulch. Such an environment is likely to be more conducive for termite survival and growth as it provides a more stable environment in which termites can break down and mix the organic fertilizer inputs using their saliva, excrete, and faecal pellets. Refs. [34–36] have reported in earlier studies that such environments are ideal for termite populations to thrive.

The generally low termite abundance in the conventional farming systems could also be attributed to the synthetic pesticides applied. Other authors found that synthetic pesticide can be highly effective [37, 38], but also varies depending on the applied management practices [39, 40]. In the current trial effectiveness of the synthetic pesticides was also generally rated as varied i.e. being effective over vegetative into early maturity of maize crop but fairly ineffective during later stages. However, we generally observed that termite abundance decreased in both high input system (conventional and organic) after pesticide specifically against termites (Dragnet, Concord and *Metarhizium anisopliae*) were applied. The *Metarhizium anisopliae* fungus in the Org-High system seemed to be effective in controlling termites as shown also by other authors [41]. Despite the use of pesticide in both high input system, termite abundance was always higher in the organic system—before and after the application. However, chemical pesticides can have severe side effects on farmers health or ecosystem functions [42, 43]. Bio-pesticides like botanicals or biological control agents could be environmental friendly and low-risk alternatives [44–47].

Overall, the recorded termite diversity in the Org-High system is similar to results in studies from Zimbabwe [48] and Nigeria [49], where 7 and 10 genera (respectively) were found in agricultural fields. Our study corroborates the observation of comparatively low termite diversity in agro-ecosystems and confirms the hypotheses that termites are not resilient towards pronounced anthropogenic disturbance [50]. Termite diversity was found to be higher in less disturbed ecosystem as shown by [49] who found 19 and 15 termite genera in the primary forest and disturbed forest, respectively. Yet, the finding of significantly higher termite diversity in the organic high-input system demonstrates that farming practices such as applying compost, mulch and cover crops as well as irrigation can mitigate the negative effects of farming on termite diversity. In addition, these farming practices can have a positive effect on soil quality (see introduction and [14–21]), and might contribute to an improved productivity of maize crop in organic high-input system [28, 29].

However, a detailed study on yield losses/gains due to termite presence and activity was not done. Generally, since the beginning of the experiment yields of French beans in Org-High system were lower, but baby corn yields were similar or even higher to conventional systems [29]. During the study period, however, we found lower yields of baby corn in Org-High compared to Conv-High at Thika (~ 10%), which could be attributed to termite presence. The generally lower rainfalls at Thika could have most likely affected the availability of other food sources in the environment and thus termite colonies have to attack and forage on the crops grown in the plots for food given the dry spells. The preferred environment for termite activity was only given in the Org-High plots (see above), thus termite were searching for food there. This is also confirmed by our field observation and other authors noticing links between decreasing vegetation cover and crops becoming more susceptible to termite damage [51, 52]. However, we cannot make a direct yield comparison from low input system to the baby corn yield in the Org-High because in the low input system a maize/bean intercrop was grown. Nonetheless, other authors could show positive links between termite presence and yield: [53] showed an yield increase of 36% in yield, which they suggest happen due to the improved soil water infiltration and improved soil nitrogen. However, due to experimental design (system experiment) it is only partially possible to link yield losses/gains to termite presence, because several factors influencing this parameter. Nonetheless, further studies on termite crop damage and associated yield losses are necessary.

### The effect of soil depths, trial sites and cropping seasons on termite abundance, incidence index and activity

The large differences in termite abundance and incidence between the two sites (Chuka and Thika) can be explained by their geographical and agro-climatic differences. Chuka, lying in semi-humid climate and receiving more annual precipitation (1500–2400 mm), is likely to offer more favorable conditions for termite populations to thrive than Thika, which lies in sub-humid climate zone and receives between 900 and 1100 mm of rainfall, experiencing something of a moisture deficit, which would negatively affect termite’s survival and reproduction rate. The absence of some genera (*Allodontotermes* and *Ancistrotermes*) at Thika might be also attributed to this. Other studies have made similar observations of termites showing that dry environments make them more vulnerable to desiccation and exposure [54, 55].

The differences in soils at the two sites might be another factor. Chuka has predominately clay and silt rich soils, whereas those at Thika have a lower clay content, another possible reason why Chuka supports higher termite populations. Refs. [56–58] have highlighted the importance of differences in soil texture on termite populations, which thrive better in clay and silt soils that optimize the termite’s biological and chemical processes [24].

The abundance and incidence of termites in the substrate was also site dependent: At Thika there were more termites found in the substrate than at Chuka. Closer observation suggests that this could be related to the fairly large numbers of predatory ants found to be feeding on termites in the soil substrates at Chuka. This could have significantly lowered termite abundance in the substrate, especially as these ants were not observed at Thika in any of the three seasons. There was also a notable decline in termite abundance between the top and sub-soil, particularly at Thika. This could be because the top soil at Thika is shallow and bulky and underlain by a hard-pan subsoil. This soil structure is likely to influence both the organic matter content along soil profile and the moisture content which would further affect termite abundance, incidence and activity throughout the soil profile.

Termites often work intensively along the soil profile while foraging for food, thus creating galleries. We found that the incidence of galleries linearly and significantly declined between the soil profiles. This could be due to the lower food availability at lower soil profiles. Refs. [59–61] have come to similar findings. Termites generally prefer cellulose and ligneous materials which they initially shred at or near the soil surface, later transporting the broken-down materials deeper into the soil profiles where they shred them further, resulting in several galleries in the residue and soil levels. These galleries increase soil porosity as they create pathways for water to percolate deeper into the soil, and these were more evident at Chuka than Thika, probably due a higher termite abundance at Chuka. Refs. [20, 62] report similar findings of greater numbers of poked holes in plots that are rich in organic material: due to the termites physically poking the soil structure. The higher moisture content of the soil at Chuka was a further biophysical characteristic difference that may have enhanced the presence of galleries at that site: a conclusion that is in line with other studies [24, 63, 64].

When looking at the seasonal effects we noted that peak termite abundance occurred at both sites during the 2nd season, and was lower in the 1st and 3rd seasons. Our hypothesis for this lies in the crops grown in each season. During the 2nd season, with higher termite abundance, a predominately pure bean crop with a closed canopy was grown which may have been more favorable to termites as the less exposed soil surface would be better at retaining moisture, which is conducive for termite growth (see above).

## Conclusions

The abundance of termites and castes, their incidence, foraging activities and diversity varied markedly between the conventional and organic farming systems. Termites, many of which are well known for their beneficial ecological roles, preferred the Org-High plots to the others. These other farming systems received no or far fewer organic inputs and lacked soil cover. These results suggest that the availability of organic matter and soil moisture in plots, along soil profiles could be one of the main determinants of termite abundance, activity and diversity. The findings demonstrates that farming practices such as applying compost, mulch and cover crops as well as irrigation can (partially) offset the disturbing effect that agriculture has on termite presence and provide a (relatively) attractive habitat for termites which, in turn, often have a beneficial effect on soil quality.

## Materials and methods

### Field sites

**Fig. 3:**
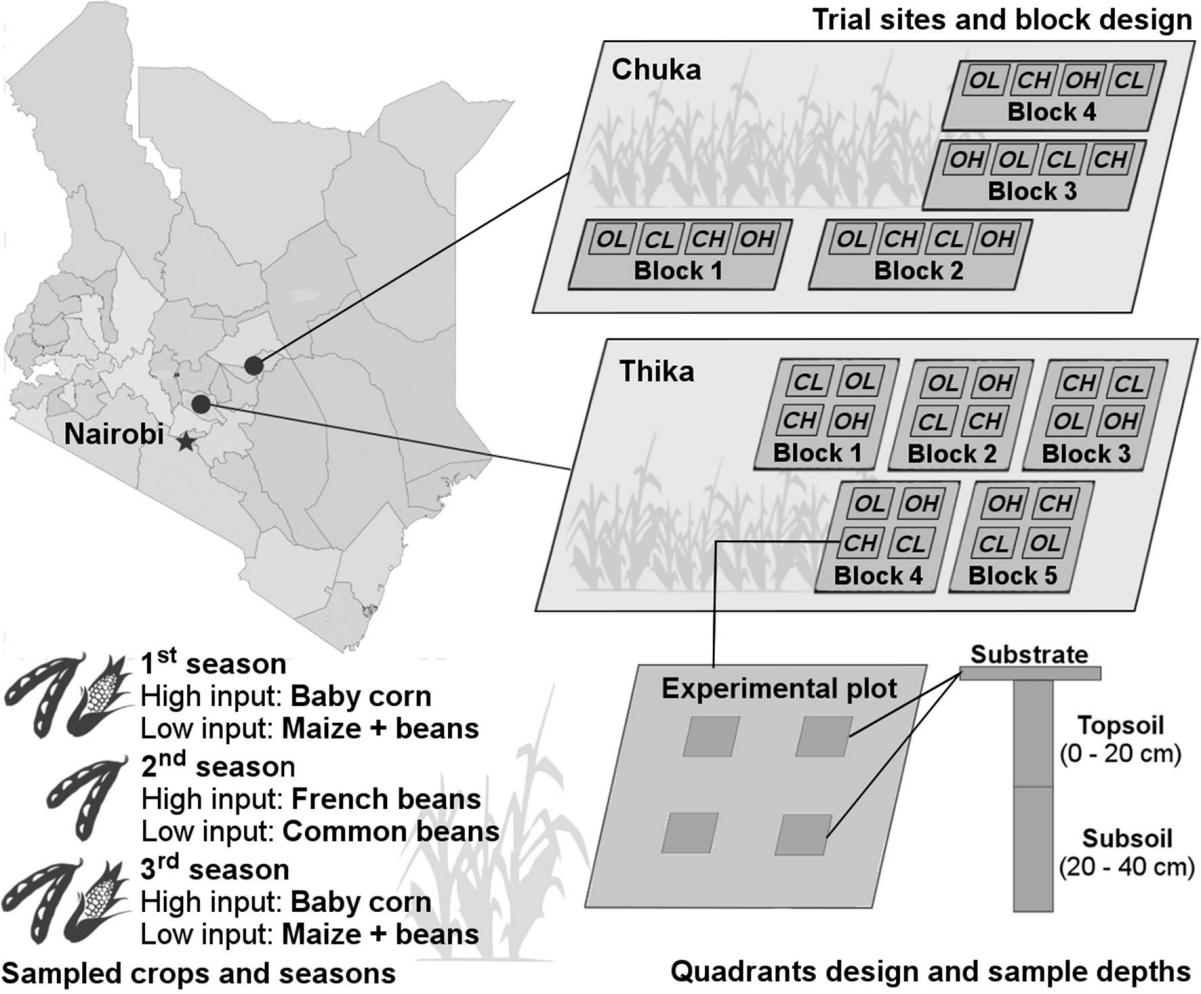
The Farming Systems Comparison Trials in Kenya (SysCom). The trial sites, block design, sampled crops, cropping seasons, quadrant design and sample depths for the termite study in the long-term experiment at Chuka and Thika, the Central Highlands of Kenya (county map is derived and adapted from http://www.opendata.go.ke)

The study was conducted between March 2014 and September 2015 in the ongoing Long-term Farming Systems Comparisons (SysCom) trials, situated in the sub-humid zones of the Central Highlands of Kenya (Fig. 3) at Chuka (Tharaka Nithi County, longitude 037° 38.792′ N and Latitude 00° 20.864′ S) and at Thika (Murang’a County, longitude 037° 04.747′ N and latitude 01° 00.231′ S). The two sites are situated in the upper midland 2 (UM_2_) and upper midland 3 (UM_3_) agro ecological zones which are described by [65] as main coffee and sunflower-maize zones, respectively. The areas are characterized by a bimodal rainfall pattern (a long rainy season from March to June and a short one from October to December) giving a mean annual rainfall of 1500 mm at Chuka and 900–1100 mm at Thika. The mean annual temperature ranges are from 19.2 to 20.6 °C at Chuka and 19.5–20.7 °C at Thika. Based on the FAO world reference base for soil resources, the soils at Chuka are Humic Nitisols while those at Thika are Rhodic Nitisols [66–68].

### Experimental design

At each site, the trial compares conventional (Conv) and organic (Org) farming at two levels of inputs: high inputs (High) representing commercial large scale production and, low inputs (Low) representing small holder production, largely for subsistence use. The management practices of these four farming systems were applied on experimental plots of 8 × 8 m (64 m^2^; net plot 6 × 6 m^2^) arranged in a Randomized Complete Block Design (RCBD), replicated four times in Chuka and five times in Thika. The termite study focused on the 1st season of 2014 (baby corn and maize-beans intercrop), the 2nd season of 2014 (French and common beans) and the 1st season of 2015 (baby corn and maize-beans intercrop). Details of field layout, crops grown, varieties, fertilizer and pest management inputs are summarized in Table 2 and graphically summarized in Fig. 3. Nonetheless, it has to be noted that pest and disease management and especially the termite control methods were different depending on site and system. At Chuka, no pesticide was used in all the systems and seasons to directly control termites. At Thika, we used the pesticides Dragnet (Pyrethroid–Permethrin; ~ 20 mL in 5 L; applied once) and Concord (Pyrethroid–Cypermethrin; ~ 4 mL in 2 L water; applied twice) to purposely control termites in the conventional systems in the first season of 2014 and 2015, respectively. In the organic systems, we used icipe formulation no. 30 (fungus *Metarhizium anisopliae*) with different carrier materials (liquid: corn oil; solid: rice) to control termites in the same seasons. The formulation was used once in 2014 (2 kg solid carrier) and twice in 2015 (1.7 kg solid carrier as well as 4 and 5 mL liquid carrier in Org-Low and Org-High, respectively). Nonetheless, other pesticides were used during the study period to control pest and disease, and some of them have ingredients which also could influence termite behavior: Bestox (Pyrethroid), Bulldock (Pyrethroid), Folicur (Tebuconazole), Dynamic (Abamectin), Thiovit (Sulphur), Ortiva (Azoxystrobin), Duduthrin (Pyrethroid), and Rodazim (Carbendazim) in Conv-High, Halt (Bacillus thuringiensis), Fosphite (Potassium Phosphite), GC3 (garlic extract), Pyerin (Pyrethrum extract), Pyegar (Pyrethrum and garlic extract)), Nimbecidine (Neem-based) and Achook (Neem-based) in Org-High, and wood ash in low input systems.

**Table 2:** The details on fertility, pest and water management of the farming systems

Farming system	Year	Season	Crop	Fertilizer management	Total N applied (kg ha^−1^)	Total P applied (kg ha^−1^)	Pest and disease management	Water management
Conv-Low	2014	LS	Maize (*Zea mays* var. H513)/Beans (*Phaseolus vulgaris* var. GLP 92)	5 t ha^−1^ of fresh FYM, 50 kg ha^−1^ DAP	31	18	Synthetic pesticides	Rain fed
	2014	SS	Common beans (*Phaseolus vulgaris* var. GLP 92)	No fertilizer application	NA	NA		
	2015	LS	Maize (*Zea mays* var. H513)/Beans (*Phaseolus vulgaris* var. GLP 92)	5 t ha^−1^ of fresh FYM, 50 kg ha^−1^ DAP	31	18		
Org-Low	2014	LS	Maize (*Zea mays* var. H513)/Beans (*Phaseolus vulgaris* var. GLP 92)	5 t ha^−1^ FYM-based compost, 100 kg ha^−1^ RP, 136 kg ha^−1^ Tithonia mulch	31	18	Biological pesticide	Rain fed
	2014	SS	Common beans (*Phaseolus vulgaris* var. GLP 92)	No fertilizer application	NA	NA		
	2015	LS	Maize (*Zea mays* var. H513)/Beans (*Phaseolus vulgaris* var. GLP 92)	5 t ha^−1^ FYM-based compost, 100 kg ha^−1^ RP, 136 kg ha^−1^ Tithonia mulch	31	18		
Conv-High	2014	LS	Baby corn (*Zea mays* var. Pannar 14)	113 t ha^−1^ FYM, 200 kg ha^−1^ DAP, 100 kg ha^−1^ CAN	113	60	Synthetic pesticides	Irrigation
	2014	SS	French beans (*Phaseolus vulgaris var.* Serengeti)	75 t ha^−1^ FYM, 200 kg ha^−1^ DAP, 100 kg ha^−1^ CAN	113	60		
	2015	LS	Baby corn (*Zea mays* var. Pannar 14)	113 t ha^−1^ FYM, 200 kg ha^−1^ DAP, 100 kg ha^−1^ CAN	113	60		
Org-High	2014	LS	Baby corn (*Zea mays* var. Pannar 14)/*Mucuna pruriens*	113 t ha^−1^ FYM-compost, 364 kg ha^−1^ RP, 54 t ha^−1^*Tithonia* mulch and 39 t ha^−1^*Tithonia* tea	113	60	Biological pesticide	Irrigation
	2014	SS	French beans (*Phaseolus vulgaris var.* Serengeti)	113 t ha^−1^ FYM-compost, 364 kg ha^−1^ RP, 54 t ha^−1^*Tithonia* mulch and 39 t ha^−1^*Tithonia* tea	113	60		
	2015	LS	Baby corn (*Zea mays* var. Pannar 14)/*Mucuna pruriens*	113 t ha^−1^ FYM-compost, 364 kg ha^−1^ RP, 54 t ha^−1^*Tithonia* mulch and 39 t ha^−1^*Tithonia* tea	113	60		

The treatment details and the cropping pattern of the long-term farming systems comparisons trials at Chuka and Thika, the Central Highlands of Kenya ([29], modified); NB: Compost preparation starts with the indicated amount of FYM and was applied at planting; CAN was applied as top-dressing in two splits; Tithonian mulch was applied after crop germination as starter N; The organic high input system also received maize stover and Mucuna intercropped with baby corn in the 1st season of 2014 and 2015 which was uprooted after harvest and incorporated following season; Assumptions: FYM/compost (DW): 1.12% total N and 0.3% P; The DM of FYM is assumed to be 40%; Tithonia diversifolia (DW): 3.3% N; 0.31% P; 3.1% K; DM of Tithonia = 20%; Phosphate rock from West Africa: 11–13% P

*Conv-Low* conventional low input farming system, *Org-Low* organic low input farming system, *Conv-High* conventional high input farming system, *Org-High* organic high input farming system, *LS* long rain season, *SS* short rain seasons, *CAN* calcium ammonium nitrate, *DAP* di-ammonium phosphate, *TSP* triple superphosphate, *RP* rock phosphate, *FYM* farm yard manure

## Data Collection

### Termite sampling and identification

A weekly termite sampling was carried every season from the 1st week after emergence (WAE) of the crop to the last harvesting day. Sampling was done in 4 quadrants within each experimental plot. Termites were sampled at different depths: (i) in the crop residue/litter on the soil surface (100 × 100 cm; substrate); and (ii) in 10 × 10 × 10 cm monolith soil profiles at different soil depths of 0–20 (topsoil) and 20–40 cm (subsoil). Caste affiliation (worker, soldier, immature) of all sampled termites and genus of the sampled termite soldiers were determined in the field as much as possible by morphological assessments using a hand lens. Subsequently, the identification of soldier to genus level was confirmed at the Nairobi National Museum using standard determination keys [69, 70]. The termites’ foraging activity was assessed in every quadrant through (i) the length of tunneled soil surfaces and substrate (cm per 10,000 cm^2^) and (ii) through the number of pocked holes/galleries at different top and subsoil (poked holes per 1000 cm^3^).

### Statistical data analysis

After finishing sampling, over 24,400 data sets on the abundance of the total number of termites, termite castes and genera and on termite activity were entered into a database and validated (checked for double or missing entries). Each data set included information about the trial site, sampling season and date, block and plot number, farming system, quadrant number and sampling depth. The abundance data was used to calculate termite incidence per quadrant expressed as (a) the presence of termites (abundance > 0) = 1, and (b) the absence of termites (abundance = 0) = 0. Afterwards, all data on termite abundance and incidence per quadrant was summarized for each plot (substrate: 40,000 cm^2^; soil: 4000 cm^3^). The incidence data was then calculated as an incidence index ranging from 0 to 4 (0% presence to 100% presence in each plot). To characterize the diversity of termite (soldier) genera we used the software EstimateS [71] to determine species richness (S), the incidence-based coverage estimator of species richness (ICE), the Chao2 estimator of species richness, the Shannon index (S_h_) and inverse Simpson index (S_i_) as diversity measures.

Data sets were separated by sample depths prior to statistical analysis. One data set included data for abundance, the incidence index, tunneling activity and diversity measures in the substrate (expressed as per 40,000 cm^2^ soil surface), and the second data set included data for abundance, the incidence index, gallery activity and diversity measures in the top and subsoil (expressed as per 4000 cm^3^ soil volume). All data sets were analyzed using R statistical software version 3.2.5 [72]. Data was analyzed with a linear mixed effect model to determine the significant effects of the fixed factors using the *lmer* function from the *lme4* package [73]. The model included 3 or 4 fixed factors: farming systems, cropping season, trial site and sampling depth (only for data relating to the top and subsoil) and their interactions, and one random factor (field replication—block). Computation of the estimated marginal means was done using the *emmeans* package [74], followed by mean separation using the adjusted Tukey’s method using the *multicompView* package for *cld* function [75]. The correlation between termite castes and genera and between foraging activities was tested using the *rcorr* function from the *Hmisc* package [76]. The significance level for all tests was α = 0.05.

## Supplementary information

**Additional file 1: Table S1.** The average abundance, incidence and activity of termites. **Table S2.** Diversity measures for termite genera.

## Data Availability

The datasets used and analyzed during the current study are available from the corresponding author on reasonable request. As we are working on a long-term experiment, we are establishing our own (SharePoint) database to store all available data. This database is not public as not all the data in the database is yet published, but the database can be accessed through a request addressing the project team at FiBL (http://systems-comparison.fibl.org/).

## Abbreviations

CAN: Calcium ammonium nitrate
Chao2: Chao’s estimator of species richness
Conv-High: Conventional high farming system
Conv-Low: Conventional low input farming system
DAP: Di-ammonium phosphate
FYM: Farm yard manure
ICE: Incidence-based coverage estimator of species richness
LS: Long rain season
na: Not applicable
ns: Not significant
Org-High: Organic high input farming system
Org-Low: Organic low input farming system
RP: Rock phosphate
S: Species richness
S_h_: Shannon index
S_i_: Inverse Simpson index
SS: Short rain seasons
TSP: Triple superphosphate
