# Supplementary material for: The impact of conventional and organic farming on soil biodiversity conservation: a case study on termites in the long-term farming systems comparison trials in Kenya
Source: BMC Ecol. 2020 Feb 27;20:13. doi: 10.1186/s12898-020-00282-x (PMC7045444; doi:10.1186/s12898-020-00282-x)
Supplement: Supplementary file 1 — Additional file 1: Table S1. The average abundance, incidence and activity of termites. Table S2. Diversity measures for termite genera. [file 12898_2020_282_MOESM1_ESM.docx]

***Table S1***

**Title:** The average abundance, incidence and activity of termites

|  |  |  |  | **Abundance** | | | | | | | |  | **Incidence** | | | | | | | |  | **Activity** | | | |
| --- | --- | --- | --- | --- | --- | --- | --- | --- | --- | --- | --- | --- | --- | --- | --- | --- | --- | --- | --- | --- | --- | --- | --- | --- | --- |
|  |  |  |  | Total | | Worker | | Soldier | | Immature | |  | Total | | Worker | | Soldier | | Immature | |  | Tunnelling | | Gallery | |
|  |  |  |  | Mean | sem | Mean | sem | Mean | sem | Mean | sem |  | Mean | sem | Mean | sem | Mean | sem | Mean | sem |  | Mean | sem | Mean | sem |
| Chuka | 1^st^ season | Substrate | Conv-Low | 0.46 | ±0.17 | 0.02 | ±0.02 | 0.00 | ±0.00 | 0.44 | ±0.16 |  | 0.31 | ±0.10 | 0.02 | ±0.02 | 0.00 | ±0.00 | 0.31 | ±0.10 |  | 1.78 | ±0.18 | NA |  |
|  |  |  | Org-Low | 0.19 | ±0.17 | 0.02 | ±0.02 | 0.00 | ±0.00 | 0.17 | ±0.15 |  | 0.10 | ±0.08 | 0.02 | ±0.02 | 0.00 | ±0.00 | 0.10 | ±0.08 |  | 1.51 | ±0.17 | NA |  |
|  |  |  | Conv-High | 1.33 | ±0.80 | 0.23 | ±0.14 | 0.10 | ±0.10 | 1.00 | ±0.58 |  | 0.31 | ±0.13 | 0.19 | ±0.11 | 0.08 | ±0.08 | 0.31 | ±0.13 |  | 1.61 | ±0.13 | NA |  |
|  |  |  | Org-High | 15.50 | ±2.46 | 2.44 | ±0.42 | 1.33 | ±0.25 | 11.73 | ±1.82 |  | 2.33 | ±0.25 | 1.63 | ±0.24 | 1.06 | ±0.19 | 2.33 | ±0.25 |  | 14.72 | ±1.36 | NA |  |
| Chuka | 1^st^ season | Topsoil | Conv-Low | 5.70 | ±0.88 | 0.89 | ±0.16 | 0.32 | ±0.08 | 4.49 | ±0.66 |  | 1.55 | ±0.15 | 0.77 | ±0.12 | 0.26 | ±0.06 | 1.55 | ±0.15 |  | NA |  | 6.80 | ±1.25 |
|  |  |  | Org-Low | 4.44 | ±0.83 | 0.63 | ±0.15 | 0.28 | ±0.07 | 3.53 | ±0.63 |  | 1.22 | ±0.14 | 0.45 | ±0.09 | 0.22 | ±0.05 | 1.20 | ±0.13 |  | NA |  | 3.27 | ±0.29 |
|  |  |  | Conv-High | 5.63 | ±0.83 | 0.85 | ±0.15 | 0.36 | ±0.09 | 4.43 | ±0.61 |  | 1.60 | ±0.16 | 0.77 | ±0.12 | 0.34 | ±0.08 | 1.60 | ±0.16 |  | NA |  | 6.09 | ±0.57 |
|  |  |  | Org-High | 32.88 | ±2.70 | 5.21 | ±0.43 | 3.45 | ±0.31 | 24.21 | ±1.99 |  | 3.03 | ±0.15 | 2.67 | ±0.16 | 2.20 | ±0.15 | 2.99 | ±0.15 |  | NA |  | 45.45 | ±5.51 |
| Chuka | 1^st^ season | Subsoil | Conv-Low | 5.17 | ±0.78 | 0.72 | ±0.14 | 0.33 | ±0.10 | 4.13 | ±0.56 |  | 1.59 | ±0.15 | 0.63 | ±0.11 | 0.26 | ±0.07 | 1.59 | ±0.15 |  | NA |  | 5.64 | ±1.31 |
|  |  |  | Org-Low | 4.40 | ±0.73 | 0.59 | ±0.13 | 0.33 | ±0.09 | 3.49 | ±0.54 |  | 1.43 | ±0.14 | 0.51 | ±0.10 | 0.27 | ±0.07 | 1.39 | ±0.14 |  | NA |  | 2.10 | ±0.21 |
|  |  |  | Conv-High | 4.27 | ±0.69 | 0.60 | ±0.13 | 0.23 | ±0.07 | 3.44 | ±0.51 |  | 1.45 | ±0.15 | 0.54 | ±0.11 | 0.21 | ±0.06 | 1.44 | ±0.15 |  | NA |  | 3.33 | ±0.33 |
|  |  |  | Org-High | 28.25 | ±2.75 | 4.62 | ±0.44 | 2.79 | ±0.28 | 20.85 | ±2.05 |  | 2.85 | ±0.16 | 2.52 | ±0.16 | 1.93 | ±0.16 | 2.78 | ±0.17 |  | NA |  | 24.57 | ±3.47 |
| Chuka | 2^nd^ season | Substrate | Conv-Low | 5.83 | ±2.25 | 1.00 | ±0.40 | 0.35 | ±0.12 | 4.48 | ±1.77 |  | 0.88 | ±0.22 | 0.58 | ±0.19 | 0.30 | ±0.10 | 0.80 | ±0.22 |  | 3.41 | ±0.63 | NA |  |
|  |  |  | Org-Low | 4.05 | ±1.79 | 0.58 | ±0.30 | 0.20 | ±0.11 | 3.28 | ±1.41 |  | 0.83 | ±0.22 | 0.35 | ±0.16 | 0.18 | ±0.09 | 0.78 | ±0.22 |  | 4.48 | ±0.87 | NA |  |
|  |  |  | Conv-High | 3.48 | ±1.50 | 0.55 | ±0.26 | 0.18 | ±0.11 | 2.75 | ±1.19 |  | 0.75 | ±0.20 | 0.43 | ±0.17 | 0.18 | ±0.11 | 0.70 | ±0.20 |  | 4.76 | ±0.86 | NA |  |
|  |  |  | Org-High | 43.98 | ±6.84 | 7.58 | ±1.19 | 1.40 | ±0.23 | 35.00 | ±5.58 |  | 3.78 | ±0.07 | 3.23 | ±0.17 | 1.28 | ±0.20 | 3.78 | ±0.07 |  | 41.74 | ±2.87 | NA |  |
| Chuka | 2^nd^ season | Topsoil | Conv-Low | 10.30 | ±1.41 | 1.69 | ±0.26 | 0.59 | ±0.13 | 8.03 | ±1.09 |  | 2.20 | ±0.19 | 1.38 | ±0.19 | 0.56 | ±0.12 | 2.06 | ±0.19 |  | NA |  | 10.89 | ±3.13 |
|  |  |  | Org-Low | 7.48 | ±1.27 | 1.14 | ±0.24 | 0.53 | ±0.13 | 5.81 | ±1.00 |  | 1.93 | ±0.16 | 0.88 | ±0.15 | 0.45 | ±0.10 | 1.73 | ±0.16 |  | NA |  | 5.71 | ±0.55 |
|  |  |  | Conv-High | 12.85 | ±1.66 | 1.90 | ±0.30 | 0.94 | ±0.19 | 10.01 | ±1.31 |  | 2.55 | ±0.17 | 1.38 | ±0.18 | 0.76 | ±0.14 | 2.40 | ±0.18 |  | NA |  | 12.38 | ±3.21 |
|  |  |  | Org-High | 70.43 | ±4.10 | 11.78 | ±0.69 | 3.58 | ±0.34 | 55.08 | ±3.36 |  | 3.96 | ±0.03 | 3.83 | ±0.07 | 2.38 | ±0.15 | 3.96 | ±0.03 |  | NA |  | 83.69 | ±5.18 |
| Chuka | 2^nd^ season | Subsoil | Conv-Low | 7.51 | ±1.37 | 1.16 | ±0.23 | 0.41 | ±0.10 | 5.94 | ±1.07 |  | 1.65 | ±0.18 | 0.94 | ±0.16 | 0.39 | ±0.09 | 1.59 | ±0.19 |  | NA |  | 7.00 | ±2.89 |
|  |  |  | Org-Low | 3.31 | ±0.68 | 0.46 | ±0.12 | 0.20 | ±0.06 | 2.65 | ±0.54 |  | 1.24 | ±0.15 | 0.43 | ±0.10 | 0.20 | ±0.06 | 1.10 | ±0.14 |  | NA |  | 2.69 | ±0.28 |
|  |  |  | Conv-High | 10.10 | ±1.47 | 1.64 | ±0.27 | 0.53 | ±0.12 | 7.94 | ±1.14 |  | 2.16 | ±0.18 | 1.25 | ±0.18 | 0.50 | ±0.11 | 2.08 | ±0.18 |  | NA |  | 8.08 | ±2.25 |
|  |  |  | Org-High | 54.96 | ±4.28 | 9.26 | ±0.76 | 2.66 | ±0.27 | 43.04 | ±3.49 |  | 3.88 | ±0.05 | 3.59 | ±0.10 | 2.01 | ±0.16 | 3.86 | ±0.06 |  | NA |  | 41.20 | ±4.18 |
| Chuka | 3^rd^ season | Substrate | Conv-Low | 2.30 | ±1.04 | 0.36 | ±0.18 | 0.06 | ±0.03 | 1.88 | ±0.84 |  | 0.44 | ±0.13 | 0.22 | ±0.09 | 0.06 | ±0.03 | 0.41 | ±0.12 |  | 1.11 | ±0.26 | NA |  |
|  |  |  | Org-Low | 2.22 | ±0.89 | 0.38 | ±0.16 | 0.09 | ±0.04 | 1.75 | ±0.70 |  | 0.41 | ±0.14 | 0.27 | ±0.10 | 0.09 | ±0.04 | 0.41 | ±0.14 |  | 0.94 | ±0.14 | NA |  |
|  |  |  | Conv-High | 1.23 | ±0.44 | 0.20 | ±0.09 | 0.09 | ±0.08 | 0.94 | ±0.35 |  | 0.42 | ±0.13 | 0.20 | ±0.09 | 0.08 | ±0.06 | 0.36 | ±0.12 |  | 1.11 | ±0.19 | NA |  |
|  |  |  | Org-High | 14.86 | ±3.88 | 2.53 | ±0.68 | 0.80 | ±0.14 | 11.53 | ±3.16 |  | 2.02 | ±0.19 | 1.22 | ±0.21 | 0.72 | ±0.12 | 1.63 | ±0.21 |  | 47.63 | ±2.19 | NA |  |
| Chuka | 3^rd^ season | Topsoil | Conv-Low | 5.91 | ±1.01 | 0.99 | ±0.18 | 0.35 | ±0.08 | 4.56 | ±0.77 |  | 1.12 | ±0.12 | 0.66 | ±0.11 | 0.29 | ±0.06 | 1.04 | ±0.12 |  | NA |  | 8.59 | ±2.33 |
|  |  |  | Org-Low | 6.45 | ±1.15 | 1.04 | ±0.20 | 0.36 | ±0.08 | 5.05 | ±0.89 |  | 1.32 | ±0.13 | 0.68 | ±0.11 | 0.33 | ±0.07 | 1.26 | ±0.13 |  | NA |  | 5.09 | ±0.48 |
|  |  |  | Conv-High | 3.30 | ±0.63 | 0.48 | ±0.11 | 0.24 | ±0.05 | 2.58 | ±0.50 |  | 0.94 | ±0.11 | 0.43 | ±0.09 | 0.23 | ±0.05 | 0.82 | ±0.11 |  | NA |  | 4.08 | ±1.72 |
|  |  |  | Org-High | 35.91 | ±3.35 | 5.79 | ±0.58 | 2.66 | ±0.21 | 27.47 | ±2.67 |  | 3.37 | ±0.09 | 2.39 | ±0.15 | 1.88 | ±0.12 | 3.04 | ±0.12 |  | NA |  | 64.06 | ±10.38 |
| Chuka | 3^rd^ season | Subsoil | Conv-Low | 5.73 | ±1.18 | 0.92 | ±0.20 | 0.27 | ±0.06 | 4.54 | ±0.94 |  | 1.13 | ±0.13 | 0.60 | ±0.11 | 0.25 | ±0.06 | 1.05 | ±0.13 |  | NA |  | 6.20 | ±2.12 |
|  |  |  | Org-Low | 6.31 | ±0.96 | 1.07 | ±0.18 | 0.38 | ±0.07 | 4.86 | ±0.73 |  | 1.36 | ±0.13 | 0.80 | ±0.11 | 0.34 | ±0.06 | 1.26 | ±0.13 |  | NA |  | 3.14 | ±0.33 |
|  |  |  | Conv-High | 4.02 | ±0.68 | 0.66 | ±0.13 | 0.26 | ±0.05 | 3.09 | ±0.52 |  | 0.97 | ±0.12 | 0.54 | ±0.10 | 0.25 | ±0.05 | 0.90 | ±0.12 |  | NA |  | 3.09 | ±1.2 |
|  |  |  | Org-High | 28.77 | ±2.41 | 4.86 | ±0.42 | 2.11 | ±0.20 | 21.80 | ±1.89 |  | 3.19 | ±0.11 | 2.50 | ±0.15 | 1.53 | ±0.12 | 2.93 | ±0.13 |  | NA |  | 20.13 | ±5.36 |
| Thika | 1^st^ season | Substrate | Conv-Low | 3.42 | ±1.4 | 0.56 | ±0.25 | 0.34 | ±0.12 | 2.52 | ±1.07 |  | 0.66 | ±0.18 | 0.42 | ±0.17 | 0.32 | ±0.11 | 0.54 | ±0.19 |  | 1.72 | ±0.33 | NA |  |
|  |  |  | Org-Low | 2.46 | ±1.13 | 0.42 | ±0.21 | 0.34 | ±0.09 | 1.70 | ±0.87 |  | 0.60 | ±0.16 | 0.30 | ±0.14 | 0.32 | ±0.08 | 0.36 | ±0.15 |  | 1.91 | ±0.43 | NA |  |
|  |  |  | Conv-High | 2.70 | ±1.00 | 0.44 | ±0.18 | 0.36 | ±0.12 | 1.90 | ±0.76 |  | 0.70 | ±0.18 | 0.32 | ±0.13 | 0.30 | ±0.09 | 0.50 | ±0.17 |  | 1.97 | ±0.48 | NA |  |
|  |  |  | Org-High | 16.40 | ±3.34 | 2.52 | ±0.54 | 2.72 | ±0.46 | 11.16 | ±2.46 |  | 2.04 | ±0.25 | 1.50 | ±0.27 | 1.64 | ±0.20 | 1.68 | ±0.26 |  | 51.47 | ±7.74 | NA |  |
| Thika | 1^st^ season | Topsoil | Conv-Low | 1.84 | ±0.71 | 0.21 | ±0.12 | 0.14 | ±0.04 | 1.49 | ±0.58 |  | 0.46 | ±0.12 | 0.13 | ±0.06 | 0.14 | ±0.04 | 0.39 | ±0.11 |  | NA |  | 2.30 | ±0.27 |
|  |  |  | Org-Low | 3.66 | ±0.82 | 0.56 | ±0.16 | 0.56 | ±0.09 | 2.54 | ±0.64 |  | 1.07 | ±0.14 | 0.43 | ±0.11 | 0.53 | ±0.08 | 0.66 | ±0.14 |  | NA |  | 2.18 | ±0.26 |
|  |  |  | Conv-High | 3.51 | ±0.92 | 0.53 | ±0.17 | 0.26 | ±0.08 | 2.72 | ±0.73 |  | 0.77 | ±0.14 | 0.39 | ±0.12 | 0.25 | ±0.07 | 0.67 | ±0.14 |  | NA |  | 7.12 | ±0.47 |
|  |  |  | Org-High | 9.29 | ±1.38 | 1.16 | ±0.24 | 2.67 | ±0.29 | 5.46 | ±0.97 |  | 2.11 | ±0.17 | 0.82 | ±0.15 | 1.70 | ±0.15 | 1.36 | ±0.18 |  | NA |  | 31.95 | ±3.62 |
| Thika | 1^st^ season | Subsoil | Conv-Low | 0.54 | ±0.39 | 0.09 | ±0.08 | 0.03 | ±0.02 | 0.42 | ±0.29 |  | 0.12 | ±0.06 | 0.05 | ±0.04 | 0.03 | ±0.02 | 0.11 | ±0.06 |  | NA |  | 0.24 | ±0.09 |
|  |  |  | Org-Low | 0.79 | ±0.3 | 0.15 | ±0.07 | 0.01 | ±0.01 | 0.63 | ±0.23 |  | 0.27 | ±0.09 | 0.15 | ±0.07 | 0.01 | ±0.01 | 0.26 | ±0.09 |  | NA |  | 0.16 | ±0.06 |
|  |  |  | Conv-High | 0.90 | ±0.39 | 0.13 | ±0.07 | 0.02 | ±0.01 | 0.75 | ±0.31 |  | 0.23 | ±0.08 | 0.10 | ±0.06 | 0.02 | ±0.01 | 0.23 | ±0.08 |  | NA |  | 2.05 | ±0.23 |
|  |  |  | Org-High | 2.96 | ±0.82 | 0.37 | ±0.14 | 0.82 | ±0.17 | 1.77 | ±0.56 |  | 0.79 | ±0.14 | 0.30 | ±0.10 | 0.63 | ±0.11 | 0.48 | ±0.12 |  | NA |  | 5.15 | ±2.08 |
| Thika | 2^nd^ season | Substrate | Conv-Low | 2.91 | ±1.49 | 0.43 | ±0.29 | 0.34 | ±0.11 | 2.14 | ±1.13 |  | 0.69 | ±0.21 | 0.23 | ±0.16 | 0.34 | ±0.11 | 0.49 | ±0.20 |  | 3.63 | ±1.25 | NA |  |
|  |  |  | Org-Low | 4.54 | ±2.07 | 0.74 | ±0.34 | 0.29 | ±0.13 | 3.51 | ±1.65 |  | 0.80 | ±0.23 | 0.49 | ±0.21 | 0.26 | ±0.10 | 0.66 | ±0.24 |  | 5.28 | ±1.53 | NA |  |
|  |  |  | Conv-High | 5.86 | ±1.68 | 1.03 | ±0.34 | 0.31 | ±0.11 | 4.51 | ±1.32 |  | 1.26 | ±0.27 | 0.74 | ±0.23 | 0.29 | ±0.10 | 1.09 | ±0.28 |  | 5.79 | ±1.79 | NA |  |
|  |  |  | Org-High | 88.77 | ±11.4 | 14.57 | ±1.94 | 6.17 | ±0.48 | 68.03 | ±9.29 |  | 3.80 | ±0.11 | 3.54 | ±0.22 | 3.17 | ±0.15 | 3.63 | ±0.19 |  | 282.33 | ±20.95 | NA |  |
| Thika | 2^nd^ season | Topsoil | Conv-Low | 2.63 | ±0.96 | 0.37 | ±0.16 | 0.30 | ±0.08 | 1.96 | ±0.75 |  | 0.66 | ±0.15 | 0.30 | ±0.12 | 0.29 | ±0.07 | 0.50 | ±0.15 |  | NA |  | 2.16 | ±0.41 |
|  |  |  | Org-Low | 3.26 | ±1.28 | 0.44 | ±0.22 | 0.50 | ±0.12 | 2.31 | ±1.02 |  | 0.87 | ±0.17 | 0.24 | ±0.10 | 0.46 | ±0.10 | 0.50 | ±0.14 |  | NA |  | 1.51 | ±0.38 |
|  |  |  | Conv-High | 6.07 | ±1.36 | 1.03 | ±0.26 | 0.43 | ±0.10 | 4.61 | ±1.07 |  | 1.39 | ±0.19 | 0.77 | ±0.19 | 0.40 | ±0.09 | 1.14 | ±0.19 |  | NA |  | 5.44 | ±0.86 |
|  |  |  | Org-High | 31.01 | ±3.59 | 4.81 | ±0.61 | 4.01 | ±0.36 | 22.19 | ±2.80 |  | 3.51 | ±0.14 | 2.53 | ±0.21 | 2.50 | ±0.16 | 3.16 | ±0.18 |  | NA |  | 56.51 | ±4.28 |
| Thika | 2^nd^ season | Subsoil | Conv-Low | 0.76 | ±0.28 | 0.06 | ±0.06 | 0.09 | ±0.04 | 0.61 | ±0.24 |  | 0.37 | ±0.11 | 0.06 | ±0.06 | 0.07 | ±0.03 | 0.30 | ±0.11 |  | NA |  | 0.34 | ±0.14 |
|  |  |  | Org-Low | 0.10 | ±0.05 | 0.00 | ±0.00 | 0.09 | ±0.04 | 0.01 | ±0.01 |  | 0.10 | ±0.05 | 0.00 | ±0.00 | 0.09 | ±0.04 | 0.01 | ±0.01 |  | NA |  | 0.01 | ±0.01 |
|  |  |  | Conv-High | 1.33 | ±0.48 | 0.19 | ±0.09 | 0.04 | ±0.02 | 1.10 | ±0.39 |  | 0.44 | ±0.13 | 0.19 | ±0.09 | 0.04 | ±0.02 | 0.41 | ±0.13 |  | NA |  | 1.30 | ±0.24 |
|  |  |  | Org-High | 8.04 | ±1.62 | 1.36 | ±0.29 | 1.17 | ±0.22 | 5.51 | ±1.17 |  | 1.63 | ±0.21 | 1.03 | ±0.20 | 0.96 | ±0.15 | 1.31 | ±0.22 |  | NA |  | 5.51 | ±1.12 |
| Thika | 3^rd^ season | Substrate | Conv-Low | 1.15 | ±0.42 | 0.11 | ±0.07 | 0.18 | ±0.05 | 0.86 | ±0.36 |  | 0.48 | ±0.13 | 0.11 | ±0.07 | 0.18 | ±0.05 | 0.32 | ±0.13 |  | 1.37 | ±0.3 | NA |  |
|  |  |  | Org-Low | 4.02 | ±1.21 | 0.69 | ±0.22 | 0.25 | ±0.07 | 3.08 | ±0.95 |  | 0.89 | ±0.18 | 0.54 | ±0.16 | 0.25 | ±0.07 | 0.77 | ±0.18 |  | 2.83 | ±0.54 | NA |  |
|  |  |  | Conv-High | 1.00 | ±0.45 | 0.15 | ±0.10 | 0.22 | ±0.06 | 0.63 | ±0.36 |  | 0.42 | ±0.11 | 0.12 | ±0.07 | 0.22 | ±0.06 | 0.20 | ±0.10 |  | 1.16 | ±0.27 | NA |  |
|  |  |  | Org-High | 47.63 | ±7.83 | 7.49 | ±1.28 | 4.49 | ±0.50 | 35.65 | ±6.13 |  | 3.02 | ±0.17 | 2.25 | ±0.24 | 2.49 | ±0.16 | 2.52 | ±0.23 |  | 108.74 | ±6.42 | NA |  |
| Thika | 3^rd^ season | Topsoil | Conv-Low | 1.43 | ±0.52 | 0.22 | ±0.09 | 0.16 | ±0.04 | 1.05 | ±0.41 |  | 0.41 | ±0.09 | 0.18 | ±0.07 | 0.15 | ±0.04 | 0.30 | ±0.09 |  | NA |  | 2.85 | ±0.42 |
|  |  |  | Org-Low | 6.10 | ±1.37 | 1.03 | ±0.23 | 0.38 | ±0.09 | 4.69 | ±1.06 |  | 1.08 | ±0.14 | 0.70 | ±0.12 | 0.32 | ±0.07 | 0.98 | ±0.14 |  | NA |  | 5.02 | ±1.12 |
|  |  |  | Conv-High | 2.64 | ±0.95 | 0.45 | ±0.17 | 0.34 | ±0.07 | 1.85 | ±0.74 |  | 0.55 | ±0.10 | 0.28 | ±0.08 | 0.32 | ±0.06 | 0.35 | ±0.09 |  | NA |  | 3.12 | ±0.54 |
|  |  |  | Org-High | 30.67 | ±3.61 | 4.95 | ±0.58 | 3.25 | ±0.34 | 22.47 | ±2.73 |  | 2.65 | ±0.15 | 2.13 | ±0.16 | 1.78 | ±0.13 | 2.44 | ±0.16 |  | NA |  | 59.96 | ±4.2 |
| Thika | 3^rd^ season | Subsoil | Conv-Low | 0.75 | ±0.42 | 0.06 | ±0.04 | 0.07 | ±0.03 | 0.62 | ±0.37 |  | 0.14 | ±0.06 | 0.05 | ±0.03 | 0.06 | ±0.02 | 0.11 | ±0.05 |  | NA |  | 0.24 | ±0.09 |
|  |  |  | Org-Low | 0.41 | ±0.17 | 0.08 | ±0.04 | 0.01 | ±0.01 | 0.32 | ±0.14 |  | 0.14 | ±0.05 | 0.08 | ±0.04 | 0.01 | ±0.01 | 0.13 | ±0.05 |  | NA |  | 0.30 | ±0.08 |
|  |  |  | Conv-High | 0.78 | ±0.37 | 0.15 | ±0.07 | 0.03 | ±0.02 | 0.61 | ±0.29 |  | 0.17 | ±0.07 | 0.12 | ±0.05 | 0.03 | ±0.02 | 0.15 | ±0.06 |  | NA |  | 0.86 | ±0.18 |
|  |  |  | Org-High | 8.87 | ±1.85 | 1.45 | ±0.31 | 0.93 | ±0.17 | 6.49 | ±1.38 |  | 1.12 | ±0.15 | 0.74 | ±0.13 | 0.63 | ±0.10 | 1.02 | ±0.15 |  | NA |  | 11.20 | ±2.46 |
| **Source of variation for substrate** | | | |  | |  | |  | |  | |  |  | |  | |  | |  | |  |  | |  | |
| Farming system | | | | *** | | *** | | *** | | *** | |  | *** | | *** | | *** | | *** | |  | *** | | na | |
| Season | | | | *** | | *** | | *** | | *** | |  | *** | | *** | | *** | | *** | |  | *** | | na | |
| Site | | | | ** | | ** | | *** | | ** | |  | * | | ** | | *** | | ns | |  | *** | | na | |
| Farming system x season | | | | *** | | *** | | *** | | *** | |  | *** | | *** | | *** | | *** | |  | *** | | na | |
| Farming system x site | | | | *** | | *** | | *** | | *** | |  | ns | | ns | | *** | | ns | |  | *** | | na | |
| Farming system x site x season | | | | *** | | *** | | *** | | *** | |  | ** | | ** | | *** | | *** | |  | *** | | na | |
| **Source of variation for soil** | | | |  | |  | |  | |  | |  |  | |  | |  | |  | |  |  | |  | |
| Farming system | | | | *** | | *** | | *** | | *** | |  | *** | | *** | | *** | | *** | |  | na | | *** | |
| Depth | | | | *** | | *** | | *** | | *** | |  | *** | | *** | | *** | | *** | |  | na | | *** | |
| Season | | | | *** | | *** | | *** | | *** | |  | *** | | *** | | *** | | *** | |  | na | | *** | |
| Site | | | | *** | | *** | | ** | | *** | |  | *** | | *** | | *** | | *** | |  | na | | ** | |
| Farming system x depth | | | | *** | | *** | | *** | | *** | |  | *** | | *** | | *** | | *** | |  | na | | *** | |
| Farming system x season | | | | *** | | *** | | * | | *** | |  | *** | | *** | | *** | | *** | |  | na | | *** | |
| Farming system x site | | | | *** | | *** | | *** | | *** | |  | *** | | *** | | *** | | *** | |  | na | | *** | |
| Farming system x depth x season | | | | ** | | ** | | ns | | ** | |  | ns | | ns | | ns | | ns | |  | na | | *** | |
| Farming system x site x depth | | | | * | | * | | *** | | ns | |  | *** | | *** | | *** | | *** | |  | na | | ns | |
| Farming system x site x season | | | | *** | | *** | | *** | | *** | |  | ** | | *** | | *** | | ns | |  | na | | ** | |
| Farming system x depth x season x site | | | | ns | | ns | | ns | | ns | |  | ns | | * | | ns | | * | |  | na | | ns | |

***Legend:*** *The average, standard error of means and sources of variation for average termite abundance, incidence index, tunneling and gallery activity for the total number of termites and termite caste in the top- and subsoil and in the substrate in organic and conventional farming systems in the farming systems comparisons trials at Chuka and Thika, the Central Highlands of Kenya; na, not applicable; ns, not significant; NB: Significant differences between farming system, site, season, depth or their interactions are indicated by * (p < 0.05), ** (p <0.01) or *** (p < 0.001); Specifications (fixed and random factors) of the linear model can be found in the chapter “Methods”*

***Table S2***

**Title:** Diversity measures for termite genera

|  |  |  |  | **S** | | **ICE** | | **Chao2** | | **S_h_** | | **S_i_** | |
| --- | --- | --- | --- | --- | --- | --- | --- | --- | --- | --- | --- | --- | --- |
|  |  |  |  | Mean | sem | Mean | sem | Mean | sem | Mean | sem | Mean | sem |
| Chuka | 1^st^ season | Substrate | Conv-Low | 0.66 | ±0.04 | 0.66 | ±0.04 | 0.66 | ±0.04 | 0.00 | ±0.00 | 1.00 | ±0.00 |
|  |  |  | Org-Low | 0.54 | ±0.06 | 0.51 | ±0.06 | 0.51 | ±0.06 | 0.01 | ±0.00 | 1.02 | ±0.00 |
|  |  |  | Conv-High | 1.00 | ±0.13 | 1.68 | ±0.33 | 1.68 | ±0.33 | 0.30 | ±0.07 | 1.50 | ±0.12 |
|  |  |  | Org-High | 4.32 | ±0.19 | 6.47 | ±0.28 | 4.87 | ±0.2 | 0.97 | ±0.03 | 2.11 | ±0.04 |
| Chuka | 1^st^ season | Topsoil | Conv-Low | 2.73 | ±0.16 | 4.47 | ±0.25 | 3.06 | ±0.16 | 0.54 | ±0.04 | 1.53 | ±0.05 |
|  |  |  | Org-Low | 3.26 | ±0.17 | 6.55 | ±0.33 | 4.45 | ±0.25 | 0.69 | ±0.03 | 1.65 | ±0.03 |
|  |  |  | Conv-High | 2.83 | ±0.13 | 4.29 | ±0.22 | 3.05 | ±0.14 | 0.58 | ±0.04 | 1.59 | ±0.06 |
|  |  |  | Org-High | 6.84 | ±0.17 | 8.32 | ±0.25 | 7.57 | ±0.21 | 1.57 | ±0.02 | 4.09 | ±0.04 |
| Chuka | 1^st^ season | Subsoil | Conv-Low | 3.61 | ±0.25 | 8.49 | ±1.00 | 5.93 | ±0.77 | 0.61 | ±0.03 | 1.54 | ±0.03 |
|  |  |  | Org-Low | 3.63 | ±0.27 | 6.74 | ±0.51 | 4.66 | ±0.40 | 0.63 | ±0.05 | 1.60 | ±0.06 |
|  |  |  | Conv-High | 2.71 | ±0.12 | 5.02 | ±0.29 | 3.34 | ±0.18 | 0.47 | ±0.02 | 1.40 | ±0.02 |
|  |  |  | Org-High | 6.92 | ±0.22 | 8.95 | ±0.32 | 7.91 | ±0.24 | 1.56 | ±0.03 | 4.02 | ±0.08 |
| Chuka | 2^nd^ season | Substrate | Conv-Low | 2.06 | ±0.20 | 3.12 | ±0.33 | 2.44 | ±0.24 | 0.63 | ±0.05 | 1.79 | ±0.07 |
|  |  |  | Org-Low | 1.60 | ±0.17 | 2.97 | ±0.50 | 2.65 | ±0.51 | 0.42 | ±0.07 | 1.61 | ±0.12 |
|  |  |  | Conv-High | 1.29 | ±0.10 | 1.84 | ±0.20 | 1.62 | ±0.19 | 0.32 | ±0.04 | 1.34 | ±0.04 |
|  |  |  | Org-High | 4.02 | ±0.15 | 6.35 | ±0.27 | 4.62 | ±0.17 | 0.81 | ±0.02 | 1.77 | ±0.02 |
| Chuka | 2^nd^ season | Topsoil | Conv-Low | 3.45 | ±0.21 | 7.03 | ±0.55 | 4.73 | ±0.37 | 0.72 | ±0.04 | 1.76 | ±0.05 |
|  |  |  | Org-Low | 2.99 | ±0.15 | 5.62 | ±0.42 | 3.70 | ±0.23 | 0.65 | ±0.03 | 1.63 | ±0.02 |
|  |  |  | Conv-High | 3.81 | ±0.20 | 7.69 | ±0.51 | 5.37 | ±0.41 | 0.79 | ±0.04 | 1.86 | ±0.04 |
|  |  |  | Org-High | 6.12 | ±0.15 | 7.35 | ±0.24 | 6.32 | ±0.15 | 1.50 | ±0.02 | 3.75 | ±0.07 |
| Chuka | 2^nd^ season | Subsoil | Conv-Low | 3.53 | ±0.20 | 8.95 | ±0.70 | 5.45 | ±0.39 | 0.74 | ±0.04 | 1.79 | ±0.06 |
|  |  |  | Org-Low | 2.29 | ±0.14 | 4.43 | ±0.46 | 2.74 | ±0.22 | 0.42 | ±0.03 | 1.38 | ±0.03 |
|  |  |  | Conv-High | 3.31 | ±0.19 | 6.39 | ±0.56 | 4.44 | ±0.37 | 0.71 | ±0.03 | 1.72 | ±0.04 |
|  |  |  | Org-High | 6.35 | ±0.15 | 7.83 | ±0.36 | 6.57 | ±0.15 | 1.48 | ±0.03 | 3.47 | ±0.08 |
| Chuka | 3^rd^ season | Substrate | Conv-Low | 1.21 | ±0.09 | 1.73 | ±0.18 | 1.34 | ±0.10 | 0.25 | ±0.03 | 1.27 | ±0.03 |
|  |  |  | Org-Low | 1.43 | ±0.11 | 2.71 | ±0.39 | 1.91 | ±0.19 | 0.41 | ±0.03 | 1.40 | ±0.03 |
|  |  |  | Conv-High | 1.02 | ±0.08 | 1.44 | ±0.19 | 1.19 | ±0.12 | 0.17 | ±0.04 | 1.23 | ±0.05 |
|  |  |  | Org-High | 3.72 | ±0.16 | 6.18 | ±0.37 | 4.29 | ±0.22 | 0.83 | ±0.02 | 1.91 | ±0.03 |
| Chuka | 3^rd^ season | Topsoil | Conv-Low | 3.35 | ±0.14 | 5.89 | ±0.26 | 4.05 | ±0.16 | 0.77 | ±0.02 | 1.86 | ±0.04 |
|  |  |  | Org-Low | 3.18 | ±0.16 | 5.05 | ±0.31 | 3.93 | ±0.25 | 0.70 | ±0.03 | 1.75 | ±0.04 |
|  |  |  | Conv-High | 3.11 | ±0.14 | 5.19 | ±0.27 | 3.94 | ±0.20 | 0.70 | ±0.02 | 1.72 | ±0.02 |
|  |  |  | Org-High | 6.89 | ±0.18 | 8.96 | ±0.22 | 7.56 | ±0.18 | 1.48 | ±0.02 | 3.51 | ±0.04 |
| Chuka | 3^rd^ season | Subsoil | Conv-Low | 3.05 | ±0.16 | 6.73 | ±0.50 | 4.36 | ±0.28 | 0.67 | ±0.02 | 1.65 | ±0.02 |
|  |  |  | Org-Low | 4.34 | ±0.18 | 8.00 | ±0.39 | 5.31 | ±0.22 | 0.89 | ±0.03 | 1.95 | ±0.05 |
|  |  |  | Conv-High | 3.42 | ±0.16 | 7.05 | ±0.43 | 4.94 | ±0.27 | 0.77 | ±0.02 | 1.80 | ±0.02 |
|  |  |  | Org-High | 6.78 | ±0.18 | 8.72 | ±0.25 | 7.33 | ±0.18 | 1.46 | ±0.02 | 3.35 | ±0.04 |
| Thika | 1^st^ season | Substrate | Conv-Low | 1.86 | ±0.17 | 4.03 | ±0.50 | 3.26 | ±0.46 | 0.68 | ±0.07 | 1.96 | ±0.11 |
|  |  |  | Org-Low | 1.61 | ±0.16 | 2.40 | ±0.30 | 1.82 | ±0.19 | 0.47 | ±0.06 | 1.63 | ±0.08 |
|  |  |  | Conv-High | 1.79 | ±0.22 | 3.20 | ±0.55 | 2.34 | ±0.36 | 0.43 | ±0.07 | 1.69 | ±0.12 |
|  |  |  | Org-High | 4.34 | ±0.20 | 6.23 | ±0.34 | 5.02 | ±0.22 | 1.21 | ±0.03 | 3.12 | ±0.08 |
| Thika | 1^st^ season | Topsoil | Conv-Low | 1.66 | ±0.14 | 2.59 | ±0.29 | 1.88 | ±0.17 | 0.44 | ±0.05 | 1.51 | ±0.06 |
|  |  |  | Org-Low | 2.73 | ±0.15 | 4.18 | ±0.25 | 3.19 | ±0.21 | 0.74 | ±0.04 | 1.93 | ±0.07 |
|  |  |  | Conv-High | 2.20 | ±0.25 | 3.74 | ±0.50 | 2.93 | ±0.42 | 0.52 | ±0.06 | 1.66 | ±0.09 |
|  |  |  | Org-High | 4.48 | ±0.18 | 6.04 | ±0.28 | 4.92 | ±0.2 | 1.24 | ±0.03 | 3.21 | ±0.08 |
| Thika | 1^st^ season | Subsoil | Conv-Low | 1.16 | ±0.20 | 2.28 | ±0.59 | 1.51 | ±0.31 | 0.28 | ±0.07 | 1.33 | ±0.09 |
|  |  |  | Org-Low | 0.89 | ±0.07 | 1.00 | ±0.10 | 0.93 | ±0.08 | 0.07 | ±0.02 | 1.06 | ±0.02 |
|  |  |  | Conv-High | 0.88 | ±0.09 | 1.30 | ±0.25 | 0.97 | ±0.12 | 0.14 | ±0.04 | 1.13 | ±0.04 |
|  |  |  | Org-High | 3.55 | ±0.19 | 6.95 | ±0.63 | 5.28 | ±0.35 | 1.20 | ±0.03 | 3.12 | ±0.08 |
| Thika | 2^nd^ season | Substrate | Conv-Low | 1.60 | ±0.18 | 2.50 | ±0.36 | 2.04 | ±0.27 | 0.57 | ±0.07 | 1.79 | ±0.10 |
|  |  |  | Org-Low | 1.58 | ±0.20 | 3.37 | ±0.78 | 2.39 | ±0.41 | 0.46 | ±0.06 | 1.56 | ±0.08 |
|  |  |  | Conv-High | 1.69 | ±0.18 | 2.65 | ±0.37 | 1.94 | ±0.22 | 0.40 | ±0.06 | 1.46 | ±0.07 |
|  |  |  | Org-High | 5.31 | ±0.17 | 6.79 | ±0.29 | 5.78 | ±0.2 | 1.34 | ±0.03 | 3.31 | ±0.07 |
| Thika | 2^nd^ season | Topsoil | Conv-Low | 2.40 | ±0.26 | 3.75 | ±0.50 | 2.79 | ±0.31 | 0.69 | ±0.07 | 1.93 | ±0.11 |
|  |  |  | Org-Low | 2.34 | ±0.23 | 4.00 | ±0.50 | 2.81 | ±0.29 | 0.68 | ±0.06 | 1.90 | ±0.10 |
|  |  |  | Conv-High | 2.91 | ±0.18 | 5.55 | ±0.54 | 3.72 | ±0.31 | 0.74 | ±0.05 | 1.92 | ±0.12 |
|  |  |  | Org-High | 5.67 | ±0.20 | 7.26 | ±0.36 | 5.94 | ±0.22 | 1.43 | ±0.03 | 3.65 | ±0.08 |
| Thika | 2^nd^ season | Subsoil | Conv-Low | 1.56 | ±0.16 | 2.26 | ±0.30 | 1.77 | ±0.20 | 0.42 | ±0.04 | 1.43 | ±0.04 |
|  |  |  | Org-Low | 0.57 | ±0.08 | 0.56 | ±0.08 | 0.56 | ±0.08 | 0.01 | ±0.00 | 1.01 | ±0.00 |
|  |  |  | Conv-High | 1.14 | ±0.13 | 1.61 | ±0.23 | 1.20 | ±0.13 | 0.18 | ±0.03 | 1.14 | ±0.03 |
|  |  |  | Org-High | 3.93 | ±0.24 | 6.11 | ±0.43 | 4.68 | ±0.29 | 1.05 | ±0.04 | 2.55 | ±0.09 |
| Thika | 3^rd^ season | Substrate | Conv-Low | 1.42 | ±0.10 | 2.26 | ±0.24 | 1.68 | ±0.13 | 0.47 | ±0.04 | 1.66 | ±0.06 |
|  |  |  | Org-Low | 2.29 | ±0.15 | 4.38 | ±0.37 | 3.09 | ±0.23 | 0.58 | ±0.04 | 1.66 | ±0.07 |
|  |  |  | Conv-High | 1.10 | ±0.07 | 1.38 | ±0.11 | 1.15 | ±0.08 | 0.26 | ±0.03 | 1.33 | ±0.04 |
|  |  |  | Org-High | 5.43 | ±0.12 | 6.59 | ±0.19 | 5.77 | ±0.12 | 1.43 | ±0.02 | 3.66 | ±0.06 |
| Thika | 3^rd^ season | Topsoil | Conv-Low | 2.25 | ±0.16 | 3.39 | ±0.27 | 2.80 | ±0.20 | 0.66 | ±0.03 | 1.80 | ±0.05 |
|  |  |  | Org-Low | 3.26 | ±0.19 | 5.90 | ±0.40 | 4.17 | ±0.26 | 0.76 | ±0.04 | 1.86 | ±0.06 |
|  |  |  | Conv-High | 2.54 | ±0.19 | 4.70 | ±0.65 | 3.31 | ±0.36 | 0.69 | ±0.06 | 2.14 | ±0.13 |
|  |  |  | Org-High | 6.11 | ±0.12 | 7.19 | ±0.19 | 6.41 | ±0.12 | 1.53 | ±0.02 | 3.99 | ±0.06 |
| Thika | 3^rd^ season | Subsoil | Conv-Low | 1.60 | ±0.16 | 3.46 | ±0.59 | 2.72 | ±0.36 | 0.61 | ±0.07 | 1.72 | ±0.09 |
|  |  |  | Org-Low | 0.72 | ±0.06 | 0.81 | ±0.08 | 0.76 | ±0.07 | 0.07 | ±0.02 | 1.08 | ±0.02 |
|  |  |  | Conv-High | 1.37 | ±0.14 | 2.53 | ±0.36 | 2.00 | ±0.24 | 0.45 | ±0.05 | 1.61 | ±0.09 |
|  |  |  | Org-High | 4.71 | ±0.20 | 7.70 | ±0.29 | 5.81 | ±0.19 | 1.25 | ±0.02 | 2.99 | ±0.06 |
| **Source of variation for substrate** | | | |  | |  | |  | |  | |  | |
| Farming system | | | | *** | | *** | | *** | | *** | | *** | |
| Season | | | | *** | | ns | | * | | ns | | ns | |
| Site | | | | *** | | ns | | * | | *** | | ** | |
| Farming system x season | | | | ns | | ns | | ns | | ns | | ns | |
| Farming system x site | | | | ns | | ns | | ns | | ns | | *** | |
| **Source of variation for soil** | | | |  | |  | |  | |  | |  | |
| Farming system | | | | *** | | *** | | *** | | *** | | *** | |
| Depth | | | | *** | | ns | | * | | *** | | *** | |
| Season | | | | ** | | ns | | ns | | ** | | ns | |
| Site | | | | ** | | ** | | ** | | * | | ns | |
| Farming system x depth | | | | ns | | ** | | * | | ** | | ns | |
| Farming system x season | | | | * | | * | | * | | ns | | ns | |
| Farming system x site | | | | ns | | *** | | ns | | ns | | ns | |

***Legend:*** *The average, standard error of means and sources of variation for species richness (S), the incidence-based coverage estimator of species richness (ICE), the Chao2 estimator of species richness, and the Shannon index (S_h_) and the inverse Simpson index (S_i_) in the substrate (top) and in the top- and subsoil (bottom) in the farming systems comparisons trials at Chuka and Thika, the Central Highlands of Kenya; ns, not significant; NB: Significant differences between farming system, site, season, depth or their interactions are indicated by * (p < 005), ** (p <001) or *** (p < 0001). Specifications (fixed and random factors) of the linear model can be found in the chapter “Methods”*
